# Anxiety in Older Adolescents at the Time of COVID-19

**DOI:** 10.3390/jcm9103064

**Published:** 2020-09-23

**Authors:** Pietro Smirni, Gioacchino Lavanco, Daniela Smirni

**Affiliations:** 1 Department of Educational Sciences, University of Catania, 95124 Catania, Italy; pietro.smirni@unict.it; 2 Department of Psychology, Educational Science and Human Movement, University of Palermo, viale delle Scienze, Ed.15, 90128 Palermo, Italy; gioacchino.lavanco@unipa.it

**Keywords:** COVID-19 pandemic, virus’ transmission, fear of contagion, breathing difficulty, healthy adolescents, emotion awareness, state anxiety

## Abstract

Corona Virus Disease-19 (COVID-19) is a catastrophic health risk, with psychological, emotional, social, and relational implications. From the early stages of the virus spread, the elderly population was identified as the most vulnerable, and health authorities have rightly focused on this frailer population. Conversely, less attention was given to the emotional and psychological dimensions of children and adolescents. Moreover, even though they were the subjects whose lives and health were at low risk, they, nevertheless, had to face a reality full of anxiety, fears, and uncertainties. The current study investigated the state of anxiety and emotional awareness in a sample of healthy older adolescents, 84 females and 64 males, aged 17 to 19, during the pandemic lockdown, using the Self-Rating Anxiety Scale and the Italian Emotion Awareness Questionnaire. An unexpected anxious phenomenology was found, affecting anxiety and the ideo-affective domain, while somatic symptomatology appeared to be less severe. The highest anxiety symptoms were breathing difficulties. These findings supported the hypothesis that the COVID-19 pandemic may be a risk condition for an increased state of anxiety in older adolescents and suggested the need to provide (1) an effective, empathic communication system with direct participation of older adolescents, (2) a psychological counseling service for the stress management of adolescents.

## 1. Introduction

Corona Virus Disease-19 (COVID-19) is a catastrophic health risk, with patients exhibiting symptoms that are often severe, imminent, and/or subtle, leading to very serious psychological, emotional, social, and relational implications for individuals and communities [1,2,3].

The fear of contagion has become prominent, leading some to believe that the future will be catastrophic and threatening. Fear dominates every other emotion. It is constantly reinforced by media bombardment centered on information about the dead, the number of infections, overcrowded intensive care units, hospitals unable to accommodate the sick, and other news that triggers fears and anxiety. In brief, the news can (oftentimes) spreads discord in society. Widespread panic and hyperarousal symptoms have developed. Individuals must take on a repertoire of somewhat unfamiliar behaviors to protect themselves from the virus and its lethal power. Ritualistic behaviors (with rupophobic and pathophobic imprints) are spreading—useful, perhaps, to contain the virus, but they become distressing signals of alarm and helplessness [2,4,5].

Moreover, the need to reduce the chances of contagion requires social distancing and limiting physical contact. Therefore, relationships between people experience profound changes; for example, direct communication disappears, or, in some cases, people lose the emotional value mediated by non-verbal modalities [2,4,5]. This is especially so since people have been forced to stay home for months. Meetings, places of worship and leisure, production facilities, services, schools, and universities remained closed, forcing students and teachers to experiment with new forms of ‘at distance’ teaching and learning [6,7].

Worldwide, elderly people were considered most at risk for getting COVID-19, and were more susceptible to fatal complications from the virus. Therefore, international health authorities and governments around the world directed attention and resources towards the health problems of elderly populations. Doctors, health professionals, clinical departments, and hospitals were forced to reduce their institutional interventions to respond, in the first instance, to the COVID emergency, and to treat thousands of elderly people in need of care. Requests have become increasingly demanding and health facilities are increasingly dominated by COVID patients [8].

In this frame, the world of adolescents and their psychological and emotional reactions become less focused, even if fragile individuals (such as adolescents) have high destabilizing and psychological effects. In a developmental stage, in which emotions, cognition, and peer relationships are oriented towards an expanding positive future of expectations and opportunities [9], a sudden, unpredictable, or very serious danger can compromise relationships, sociability, and any immediate and future planning [6].

A growing body of studies has investigated the psychological needs of children and adolescents during epidemics, and reported early data collected in COVID-19–affected areas in China during the outbreak [10]. Although children and adolescents seem to be less vulnerable than older adults to COVID-19, initial reports from Chinese communities reported that children and adolescents have been psychologically and socially affected, and have manifested significant behavioral disturbances [6,10]. The COVID-19 pandemic can worsen existing mental health problems, and lead to more cases among children and adolescents due to the unique combination of the public health crisis, social isolation, and economic recession [7].

In the USA, pre-COVID-19 epidemiological data reported that 35% of adolescents who received mental health services between 2012 and 2015 had turned to school mental health services [11]. Missing school for a prolonged period, therefore, could become an additional risk for the spread of mental disorders in the long-term, especially for more fragile adolescents who exclusively turn to scholastic health services for psychological and behavioral disorders [11].

Similarly, a recent study in the UK proposed a framework for prioritization relevant to psychological, social, and neuroscientific research, for mental health management during the pandemic [12]. The authors prioritize ascertaining and reducing the impact of the effects of school closures for young people seeking assistance [13,14,15]. Therefore, since adolescents are not indifferent to the dramatic impact of the COVID-19 epidemic, understanding their behaviors and emotions in response to this emergency can certainly be crucial to their psychological well-being, not only in the short-term, but in the long-term.

The literature on emotion regulation and infectious disease epidemics/pandemics highlights the importance of deepening the role of emotion regulation during these troubling times [16]. On the other hand, the impact of pandemics/global health crises, e.g., the Severe Acute Respiratory Syndrome (SARS) outbreak; Hemagglutinin Type 5 and Neuraminidase Type 1 (H5N1- Avian Influenza A) strain; Ebola virus] on the emotional and mental health of individuals has been widely articulated in literature, e.g., [17,18,19].

The current study aimed to investigate anxiety among a healthy sample of older adolescents, in order to support the need of psychological interventions with adolescents. This developmental stage may be viewed as a significant phase to assess the emotional effects of the pandemic, because it is relatively far off the emotional and behavioral complexity and instability of early adolescence, but not yet emotionally and behaviorally stabilized, as in the young adult stage [9]. The hypothesis of the study was that the COVID-19 pandemic could be a condition that leads to a greater risk of increasing the level of anxiety in healthy older adolescents, compared to the anxiety levels documented by previous literature studies in normal adolescents during non-COVID times.

## 2. Materials and Methods

### 2.1. Participants

Participants were recruited via an advertisement sent by email, in which the aims and objectives of the research were explained. Contact details of the participants were provided by school administrators who authorized the data collection.

A sample of 148 Italian students, aged between 17 and 19 (average age 17.9 ± 1.2), 84 females and 64 males, attending the last two years of high school, participated in the study.

Informed consent was obtained from adolescents over the age of 18; parental consent was required for minors to participate in the study. The study respected the anonymity and privacy of each participant, and was conducted in accordance with the Declaration of Helsinki. Moreover, 23 subjects were excluded because their questionnaires were not completed or showed some apparent inconsistencies.

### 2.2. Measures

To prevent having answers directly related to the anxious reactions to the epidemic, no measures that openly referred to the epidemic were used.

1. To evaluate anxiety, the Zung Self-Rating Anxiety Scale (SAS) was used. It is a standardized self-rating instrument, widely used in research and in clinical practices [20,21]; devised to measure state anxiety (within the previous week) as a clinical entity and an operationally defined disorder (not as a personality trait). It consists of 20 items, rated on a 1–4 Likert type scale. Subjects were asked to rate each item as to how it applied to them within the past week, in the following four quantitative terms: (1) none or a little of the time; (2) some of the time; (3) good part of the time; or (4) most or all of the time, coded respectively as 1, 2, 3, and 4. The total score ranged from 20 (no anxiety at all) to 80 (severe anxiety). Thus, the lower the score, the less anxious the subject, and vice versa.
2. To investigate emotional awareness, the following five subscales of the self-reported Italian Emotion Awareness Questionnaire (EAQ) for children and adolescents [22,23] were used: (1) differentiating emotions, as being aware of one’s emotional states and understanding them as distinct states, is composed of seven items, e.g., “it is difficult to know whether I feel sad or angry or something else”; (2) verbal sharing of emotions, as the tendency to share emotional experiences with others, is composed of four items, e.g., “I find it hard to talk to anyone about I feel”; (3) not hiding emotions, as the tendency to refuse to deal with their emotions, is composed of four items, e.g., “when I am angry or upset, I try to hide this”; (4) attending to others’ emotions, as the ability to perceive the emotions of others, is composed of five items, e.g., “it is important to know how my friends are feeling”; (5) analyses of one’s own emotions, as the ability to detect and recognize one’s own feelings, is composed of five items; e.g., “when I am angry or upset, I try to understand why”. Participants were asked to rate the degree to which each item was true about them on a 3-point scale (1: Not true; 2: Sometimes true; 3: Often true). Scores ranged from 25 to 75. The higher the score, the better the emotional awareness.

### 2.3. Methods

Due to mobility restrictions imposed by the Italian government to contain the serious spread of the epidemic, the data were collected online from 15 April to 15 May 2020, a period in which the Italian population was forced to stay home in protective isolation.

Participants were asked to answer two self-rating scales, using their PC or tablet. They were also asked for information about the socio-economic status of their families (parents’ occupation and education, geographical origin), and if they, or any of their family members, had been directly infected or exposed to a high risk of contagion.

### 2.4. Statistical Analysis

Means, standard deviations (SD), and percentages of the responses were computed for each item of the SAS and EAQ questionnaires. For each questionnaire, the items were sorted in descending order of severity.

Following Zung’s procedure, an overall SAS index was calculated by dividing the sum of the scores on the 20 items by the maximum score of 80, and multiplying by 100 [20,24]. Similarly, for each item, an SAS index was calculated, considering the maximum score of 4.

The differences between males and females were investigated using the independent *t*-test.

The Pearson correlation was used to measure the strength of the relationship between SAS and EAQ. A *p* value < 0.05 was set as an indication of statistical significance for the analyses.

## 3. Results

None of the participants had been directly affected by COVID or were at high risk of contagion. For demographic conditions, study participants were a homogeneous group. They all had a middle socioeconomic status, and came from three regions of southern Italy (Sicilia, Campania, and Calabria).

Table 1 shows the mean and standard deviation of absolute values out of a maximum score of 4, as well as the SAS index of items of anxiety severity (listed in descending order). The SAS overall mean score was 42.2 ± 4.7 with an SAS index of 52.7. In 12 out of 20 items, the mean agreement score was higher than or equal to 2 (on maximum 4), indicating that, in these items, most participants tended to choose the high anxious agreement responses “good part of the time”, coded as 3, or “most or all of the time”, coded as 4. The item recording the highest score was item number 13, concerning breathing difficulties (“I can breathe in and out easily”), with a mean of 3.4 ± 0.81 and an SAS index of 85.

Similarly, in item 19 of sleep disorder (“I fall asleep easily and get a good night’s rest”), the mean was 2.6 ± 0.10. Likewise, for items of anxiety, panic, negative expectations of the future, or somatic signals of anxiety, i.e., item 1, “I feel more nervous and anxious than usual” (mean 2.5 ± 0.68); item 3, “I get upset easily or feel panicky” (mean 2.4 ± 0.78); item 5, “I feel that everything is all right and nothing bad will happen” (mean 2.3 ± 0.79); and item 17 “My hands are usually dry and warm” (mean 2.4 ± 1.0).

On the contrary, the lowest score items referred to somatic disorders, such as item 12, “I have fainting spells or feel like it” (mean 1.4 ± 0.63), where 96% of participants had chosen the lowest scores (“none or a little of the time” coded as 1 or “some of the time” coded as 2), or item 6 “My arms and legs shake and tremble” (mean 1.4 ± 0.78), where participants tended to choose the lowest scores (1 or 2).

Table 2 displays mean values, standard deviations, and the percentage scores in the EAQ and in its individual subscales, in descending order. “Attending to others’ emotions”, “analyses of own emotions”, and “differentiating emotions” were the subscales with the highest scores, indicating high specific emotional abilities. In these subscales, the average agreement score was between two and three, showing that most participants tended to choose between “sometimes true” coded as 2 and “often true”, coded as 3.

Conversely, “verbal sharing of emotions” and “not hiding emotions” subscales reached the lowest scores, indicating a low specific emotional ability. In both scales, the average agreement score was below 2, showing that most participants tended to choose between “not true” coded as 1, or “sometimes true” coded as 2. For example, item 6 of the verbal sharing of emotions subscale: “when I am upset about something, I often keep it to myself” (mean 1.70 ± 0.68); or item 15 of the not hiding emotions’ subscale: “when I am upset, I try not to show it” (mean 1.78 ± 0.71).

Furthermore, both SAS and the EAQ scores were examined in relation to sex. Female anxiety total scale scores appeared significantly higher than those of males (43.5 ± 4.6 vs. 39.3 ± 3.7; t_146_ = 3.06; *p* = 0.003), but not EAQ scores (57.9 ± 10.4 vs. 58.3 ± 8.3; t_146_ = 0.15, *p* = 0.88).

SAS total score and EAQ total score did not correlate and the Pearson correlation index was very close to zero (r = −0.09, *p* = 0.28). Correlations between SAS total scores and individual EAQ subscales were similarly low.

## 4. Discussion

The current study aimed to investigate the state of anxiety and emotional awareness in a sample of healthy older adolescents. It was hypothesized that, because of the effects of the COVID-19 pandemic, the sample would have shown a high level of anxiety. The age group around 18 was chosen to minimize any high anxiety levels or low emotional awareness due to the emotional and affective instability in young adolescence [25,26,27]. The questionnaires SAS and EAQ were chosen because they did not reference the pandemic.

In the current study, over half of the SAS individual items reached a high anxiety score and, consequently, the SAS total score reached an unusually high anxiety score (SAS index 52.7).

Previous studies, during non-COVID times, found lower total SAS scores, both in large non-clinical samples of college students, in control subjects, and even in several groups of psychiatric patients [20,21,24,28]. For example, normal subjects of Zung’s study (*n* = 100) had a mean SAS index significantly lower than all five groups of patients examined (33.8 ± 5.9), while the patient sample (*n* = 225) reached mean indices ranging from 45.8 to 58.7. Patients with anxiety disorders showed a mean SAS index significantly higher than those of the other four diagnostic groups (58.7 ± 13.5) [20].

Similarly, in studies comparing normal controls, psychiatric patients, and subjects with anxiety disorders found in healthy groups, SAS indices ranged between 40 and 43 [21,24,28].

Moreover, the Zung rating scale measures state anxiety as a transient expression of a temporary emotional condition, relative to the current period (within the previous week). The state anxiety construct refers to a momentary interruption of an emotional positive continuum expressed in a subjective feeling of tension, worry, restlessness, nervousness, and reactivity, also through the activation of the autonomic nervous system and several physiological activations [29,30]. Conversely, the trait anxiety construct expresses a stable modality of emotional functioning dominated by anxiety, which favors a constant perception of danger and threat, even behind neutral events, or with low anxiety values. Therefore, since the sample was a healthy, non-clinical one, and the SAS measured state and non-trait anxiety, the unusually high anxiety scores observed would not appear to be attributed to the sample’s stable emotional functioning, but it is likely due to a temporary condition or feeling of tension and apprehension that favors a leavening of anxious responses.

Analyzing the single items, in the same previous studies, the item of breathing difficulties reached average scores lower than 2, both in normal subjects and in psychiatric patients [21,24,28]. Meanwhile, a study on patients with anxiety disorders [28] documented a score of 3.31 ± 0.99, very close to the score of the current studied adolescents (3.4 ± 0.81). It is interesting to point out that it is widely shared (among public opinion) that breath is a very easy vehicle for virus transmission, and coronavirus mainly affects respiratory functions, while breathing difficulties are among the first manifestations of viral activity in the human body. It is very understandable, therefore, that a high percentage of sample complaints concern not being able to “breathe in and out easily”. Moreover, breathing rhythms change in accordance to emotional stress. Anxiety, stress, or panic increase the respiratory rate and the amount of air in the lungs resulting in the feeling of shortness of breath. Chronic respiratory diseases in pediatric age appeared as a significant source of stress, also, for mothers, impacting their personality traits and memory performances [31].

Likewise, items referring to sleep disorder, anxiety, panic, and a negative expectation of the future reached high average scores. Sleep is one of the great anxiety-sensitive functions. Just as the catastrophic expectations of the future, restlessness, and feeling nervous, are symptomatic expressions of anxiety through the motor and neurovegetative pathways.

Coronavirus not only brings death in the short-term, but it also destabilizes behavior patterns in the long-term. The risk and fear of contagion have modified production models, employment policies, social and interpersonal relationships, leisure habits, education, and training systems and every consolidated behavioral repertoire—this is especially so for younger people. Therefore, the overall future becomes nebulous, confused, uncertain, and distressing. An anxious phenomenology develops, affecting anxiety and the ideo-affective dimension, while the somatic symptomatology, such as fainting, tremors, dizziness, and paresthesia, appears to be less severe.

Conversely, on the EAQ, the total emotional awareness score reached quite high levels compared to the maximum score, showing valid emotional abilities in the sample. On the qualitative view, “Attending to others’ emotions” and “Analyses of one’s own emotions” were the two subscales with the highest scores. The participants considered it important to know, analyze, understand, and care for the emotions of others, as well as their own, both in normal and problematic conditions (i.e., “if a friend is upset”). Contrarily, they self-rated as less willing to verbally share their own emotions with others, and they showed difficulty explaining emotions, i.e., “to talk to anyone about how I feel”, believing, for example, that “when I am feeling bad, it is no one else’s business”. On the one hand, therefore, there was openness and willingness to evaluate and understand the emotions of others and one’s own, on the other, less willingness to share one’s emotions with others.

In the correlational analysis, anxiety and emotional awareness overall scores appeared as two unrelated variables. Therefore, anxiety observed in the study did not seem associated with emotional awareness and management. Namely, it further confirmed the nature of state anxiety, which occurs temporarily in a particular historical condition, and the hypothesis that the epidemic promotes an increase in anxiety, even in adolescents with good awareness of their own (and others’) emotions. These findings supported the hypothesis that the COVID-19 pandemic and its following restrictive measures may be a risk condition for an increased state anxiety level in older adolescents. Widespread anxiety and fear, prolonged isolation in a restricted domestic environment, forced removal from school friends and relatives, the fear of being infected, confused or contradictory information, and the uncertainties of personal and family future likely supported an increase in anxious responses.

Therefore, the group of participants examined, despite showing a good level of emotional awareness at the EAQ, achieved quite high levels of state anxiety in the SAS, which cannot be seen as a stable emotional mode of functioning, but should be associated with the particular anxiety-inducing events during the time of COVID-19. Such data are consistent with similar (recent) studies involving college students in China, indicating that the students were troubled by anxiety concerning COVID-19, for the consequences on their studies [32], future employment [33], and in their interpersonal relationships [34,35].

According to the learned helplessness stress model [36], the COVID-19 pandemic and its following restrictive measures may be viewed as uncontrollable, leading to unpredictable helplessness conditions. Moreover, in line with the cumulative stress hypothesis [37], stressors, such as the physical and psychological problems related to the pandemic and the lockdown, may activate an excessive production of glucocorticoids and a deregulation of cortisol release, increasing, over time, the individual’s vulnerability to stress-related pathologies [38,39]. Given its high density of glucocorticoid receptors, the hippocampus appears as a structure particularly involved in stress responses, and in cumulative exposure to high levels of cortisol [40] that could have lasting effects on memory and cognitive processes [39,41,42,43].

Examining both the EAQ and SAS in relation to sex, female anxiety scale scores appeared significantly higher than those of males, whereas no significant differences were found concerning emotion awareness. It is likely that females feel the distress of the moment with greater anxiety, even though they manifest emotional awareness skills similar to that of boys. This finding was inconsistent with Cao and colleagues [1]—that male and female students in a sample of university students in China experienced similar stresses and negative emotions due to the epidemic.

Concerning psychological community implications, data suggest the need to develop intervention programs focused on the emotional and affective reactions of older adolescents [6,10]. As the pandemic is inevitable, unpredictable, and uncontrollable [44], and as the restrictive measures are the only way to contain the spread of the infection, the golden rule in addressing adolescent anxiety may be to provide 1) an effective, empathic, and reassuring communication system with the direct participation of adolescents, and 2) psychological counseling services for stress management.

In a recent editorial, in order to prevent “the disease of panic”, *The Lancet* highlighted that the COVID-19 pandemic cannot be prevented; nevertheless, providing people with accurate information “is the most effective prevention against the disease of panic” [45]. Communication aimed at older adolescents should offer them the possibility of being properly and honestly informed, and of getting out of isolation by sharing with others their fears, anxieties, and irrational beliefs. Such a communication system should not be only factual, but focused on their problems, for example, the management of any physical symptoms potentially related to the infection, the real ways of transmitting the virus, the duration of the restrictive measures, the short-term effects of the pandemic on the school year, lifestyle, leisure activities, interpersonal relationships and the economic conditions of their families, the validity of fake news, and the long-term effects on their futures and their families [46].

Likewise, psychological counseling should provide online services to cope with mental health issues due to anxiety from the pandemic or from intrafamilial interpersonal relations. According to Petersen’ suggestion, fear must be handled through “optimistic anxiety”—that is, being anxious enough “to take the advice of the authorities to heart” and optimistic enough to feel that one’s actions make a difference [47].

Effective, empathic information, and psychological counseling monitored by experienced adults (directly involving adolescents) can mitigate the anxious reactions of adolescents, and may help them to handle uncertainty and fear contextualizing individual vulnerability.

In conclusion, it is important to underline a limitation of the presented study concerning the sample size. Unfortunately, the unusual condition of forced distancing led us to use a remote data collection method, and to dedicate a large part of the work to build the online task. This has limited us in recruiting a larger sample. The data are descriptive given the sample size, and they must be contextualized to a very specific period and to a unique condition; therefore, they cannot be generalized.

## Figures and Tables

**Table 1:** Self-Rating Anxiety Scale (SAS): mean, standard deviations, and SAS index listed in descending order.

Items	Domain	Mean ± SD	SAS Index
13	breathing	3.4 ± 0.81	85
19	sleep	2.6 ± 1.0	65
1	nervous, anxious	2.5 ± 0.68	62.5
17	hands warm	2.4 ± 1.0	60
3	upset, panicky	2.4 ± 0.78	60
5	fear of future	2.3 ± 0.79	57.5
7	headache	2.3 ± 0.97	57.5
9	calm	2.3 ± 0.78	57.5
16	urinary	2.3 ± 0.83	57.5
8	weak, tired	2.1 ± 0.89	52.5
18	hot face	2.0 ± 0.92	50
10	heartbeat	2.0 ± 0.71	50
2	afraid no reason	1.9 ± 0.80	47.5
20	nightmares	1.9 ± 0.72	47.5
4	falling apart	1.9 ± 0.64	47.5
15	stomach ache	1.8 ± 0.82	45
14	paresthesia	1.8 ± 0.81	45
11	dizzy spells	1.6 ± 0.95	40
6	shake, tremble	1.4 ± 0.78	35
12	fainting spells	1.4 ± 0.63	35
1–20	overall	42.2 ± 4.7	52.7

**Table 2:** Emotion Awareness Questionnaire (EAQ) and its 5 subscales: means, standard deviations, and percentages listed in descending order.

EAQ Subscale	Mean ± SD	Percentage
4. Attending to others’ emotions	13.8 ± 1.9	92.1
5. Analyses of one’s own emotions	13.1 ± 2.2	87.3
1. Differentiating emotions	15.2 ± 3.5	72.3
3. Not hiding emotions	8.2 ± 2.1	68.2
2. Verbal sharing of emotions	7.9 ± 2.5	65.7
EAQ total score	58.2 ± 8.2	77.6
